# Robot-assisted partial nephrectomy for large complex renal cancer: step-by-step segmental artery unclamping

**DOI:** 10.1590/S1677-5538.IBJU.2022.0572

**Published:** 2022-12-18

**Authors:** Yong Huang, Junjie Cen, Yiming Tang, Haohua Yao, Xu Chen, Wei Chen, Junhang Luo

**Affiliations:** 1 Department of Urology the First Affiliated Hospital Sun Yat-sen University Guangzhou China Department of Urology, the First Affiliated Hospital, Sun Yat-sen University. Guangzhou, China;; 2 Department of Emergency the First Affiliated Hospital Sun Yat-sen University Guangzhou China Department of Emergency, the First Affiliated Hospital, Sun Yat-sen University, Guangzhou, China;; 3 Department of Urology The Second Affiliated Hospital Guangzhou Medical University Guangzhou China Department of Urology, The Second Affiliated Hospital, Guangzhou Medical University, Guangzhou, China

## Abstract

**Introduction:**

Main renal artery clamping and selective arterial clamping are two conventional devascularization methods for robot-assisted partial nephrectomy (RAPN) (1, 2). Decreasing warm ischemic (WI) time (3, 4) and improving clear surgical visualization (5) are the main surgically modifiable factors for RAPN, especially in large complex renal cancer (6). In this study, we described our surgical technique, focusing on gradual segmental artery unclamping on patients with large renal tumors.

**Material and methods:**

Two patients (R.E.N.A.L score 10 and 11) underwent RAPN with gradual segmental artery unclamping (Figures 1 and 2). The unclamping included five key steps. First, all renal segmental arteries were identified as tumor feeding vessel(s) and the vessels for normal kidney parenchyma under the guidance of CT angiography (CTA) 3-division (3D) reconstruction. Second, all segmental arteries were isolated, and the feeding one(s) should be blocked before other arteries were blocked. Third, the tumor was resected outside the pseudocapsule, and the deep resection bed was sutured for initial hemostasis. Fourth, the segmental arteries were reopened except for the tumor feeding one(s), and normal kidney parenchyma restored blood supply. And fifth, the resection bed was completely sutured, and the feeding vessel supplying the tumor was opened after the suture. Warm ischemia time (WIT) was defined as the time measured between clamping and unclamping of the renal artery. WIT1 was the time for normal kidney parenchyma and WIT2 was the time for resection area. Patient demographics, perioperative variables, and warm ischemic time were included in our study. And we presented the details of gradual segmental artery unclamping in the video.

**Results:**

In both cases, the total operation times were 215 and 130 mins for patient 1 and patient 2, respectively. WIT1 and WIT2 for patient 1 were 15 min and 33 min., and WIT1 and WIT2 for patient 2 were 21 min and 32 min, respectivelly. The maximum diameters of the masses resected were 10.8 and 7.3 cm, and surgical margins were negative. No patient had complications after operation. Preoperative and postoperative eGFR did not change significantly. Pre- and postoperative eGFR were 111 and 108 mL/min for patient 1, 91 and 83 mL/min for patient 2, respectively. Key hints for outcomes optimization during RAPN on patients with large complex renal tumors: 1) Each segmental renal artery is precised clamped before we excise the tumor, and an excellent surgical vision is essential for precising excision and shortening clamping time, 2) Other segmental renal arteries are unclamped except tumor feeding branch after suturing deep layer of parenchyma, and most normal parenchyma restores blood supply, 3) Preoperative high-resolution computed tomography angiography (CTA) and 3D reconstructive renal structure serve as a guide to clear the approach to find the tumor and segmental arteries (7, 8).

**Conclusions:**

Gradual segmental artery unclamping is feasible and efficient to excise large complex renal cancer. Compared with main renal artery clamping, it can shorten the warm ischemic time of normal parenchyma; On the other hand, compared with selective segmental arterial clamping, the technique can reduce bleeding from the deep resection bed, keep a clear surgical vision, and decrease the incidence of positive margin.
